# Transfer between hospitals as a predictor of delay in diagnosis and treatment of patients with Non-Small Cell Lung Cancer – a register based cohort-study

**DOI:** 10.1186/s12913-017-2230-3

**Published:** 2017-04-12

**Authors:** Maria Iachina, Erik Jakobsen, Anne Kudsk Fallesen, Anders Green

**Affiliations:** 1 Center for Clinical Epidemiology and Research Unit of Clinical Epidemiology, Odense University Hospital, University of Southern Denmark, Sdr. Boulevard 29, Entrance 216, ground floor East, DK-5000 Odense C, Denmark; 2 OPEN, Odense Patient data Exploratory Network, Odense University Hospital/Department of Clinical Research, University of Southern Denmark, Odense, Denmark; 3 The Danish Lung Cancer Registry, Department of Thoracic Surgery, Odense University Hospital, Odense, Denmark; 4 QuintilesIMS, Copenhagen, Denmark

**Keywords:** Lung cancer, Diagnosis, Treatment, Transfer

## Abstract

**Background:**

Lung cancer is the second most frequent cancer diagnosis in Denmark. Although improved during the last decade, the prognosis of lung cancer is still poor with an overall 5-year survival rate of approximately 12%. Delay in diagnosis and treatment of lung cancer has been suggested as a potential cause of the poor prognosis and as consequence, fast track cancer care pathways were implemented describing maximum acceptable time thresholds from referral to treatment. In Denmark, patients with lung cancer are often transferred between hospitals with diagnostic facilities to hospitals with treatment facilities during the care pathway. We wanted to investigate whether this organizational set-up influenced the time that patients wait for the diagnosis and treatment. Therefore, the objective of this study was to uncover the impact of transfer between hospitals on the delay in the diagnosis and treatment of Non-Small Cell Lung Cancer (NSCLC).

**Methods:**

We performed a historical prospective cohort study using data from the Danish Lung Cancer Registry (DLCR). All patients diagnosed with primary NSCLC from January 1st 2008 to December 31st 2012 were included. Patients with unresolved pathology and incomplete data on the dates of referral, diagnosis and treatment were excluded.

**Results:**

A total of 11 273 patients were included for further analyses. Transfer patients waited longer for treatment after the diagnosis, (Hazard ratio (HR) 0.81 (0.68–0.96)) and in total time from referral to treatment (HR 0.84 (0.77–0.92)), than no-transfer patients. Transfer patients had lower odds of being diagnosed (Odds Ratio (OR) 0.82 (0.74–0.94) and treated (OR 0.66 (0.61–0.72) within the acceptable time thresholds described in the care pathway.

**Conclusion:**

Fast track cancer care pathways were implemented to unify and accelerate the diagnosis and treatment of cancer. We found that the transfer between hospitals during the care pathway might cause delay from diagnosis to treatment as well as in the total time from referral to treatment in patients with Non Small-Cell Lung Cancer. The difference between no-transfer and transfer patients persists after adjusting for known predictors of delay.

## Background

In Denmark approximately 4 500 patients are diagnosed with lung cancer each year which makes it the second most frequent cancer form in the country. Although survival rates have improved during the last decade, the prognosis of lung cancer is still relatively poor with an overall 5-year survival of 12% [1].

In the late 1990’s, Denmark had significantly higher mortality on lung cancer compared to otherwise comparable countries [2–4]. Delay in diagnosis and treatment has been suggested as a potential explanation for the higher mortality among Danish lung cancer patients since their stage of disease seemed to be more advanced at the time of diagnosis and treatment compared to other countries [4, 5].

On this basis, The Danish National Board of Health has launched several national initiatives with the overall aim to improve the diagnostic processes as well as the treatment of lung cancer and through this increase the survival. Examples of these initiatives are the National Cancer Plans, clinical guidelines and cancer care pathways. Among other things, the pathways define the maximum time interval from referral to end of primary investigation and treatment.

In Denmark, the primary investigation and treatment of lung cancer are often performed at more than one hospital unit and often at geographically different settings. The surgical treatment of lung cancer is limited to four departments and the oncological treatment to 10 departments. The primary investigation on the other hand is carried out at 18 departments on a national basis. This means that some patients are transferred between hospitals e.g. between the diagnostic conclusion at the end of primary investigation and start of treatment.

The delay between referral and diagnosis and from diagnosis to treatment is a well-known and well researched phenomenon [6–16]. Several studies have shown delays for both diagnosis and treatment of lung cancer and also investigated and have found potential predictors for delay from both referrals to diagnosis and from diagnosis to onset of treatment. Examples of predicting variables are biological (age, stage, co-morbidity, type of treatment) [6, 16–19], psychological [7], socio-demographic (residential status, socioeconomic status) [6, 7, 20] and organizational (type of hospital, diagnosis and treatment at two or more different hospitals [6, 18, 21].

Danish data from The Danish Lung Cancer Registry (DLCR) have shown problems fulfilling the time thresholds and some patients experience longer delay than defined as acceptable in the cancer care pathways [1].

Due to the well-established and very comprehensive collection of data in the Danish national registries, we have the opportunity to investigate organizational factors that potentially could affect the delay in addition to the already known factors related to delay.

The objective of this study is therefore to investigate the significance of primary investigation and treatment at two or more hospitals on the delay in Danish patients with Non-Small Cell Lung Cancer (NSCLC). Transfer between hospitals as a predictor for delay is not commonly described in the literature. In this paper we aim to estimate the effect of transfer on the delay in diagnosis and treatment using the national based registers.

## Methods

We performed a retrospective cohort study based on register data from The Danish Lung Cancer Registry.

### Definitions

In this paper, “Transfer” refers to patients that undergo primary investigation and treatment at two or more different hospitals at different geographical locations. Conversely “No-Transfer” refers to patients that are diagnosed and treated at the same hospital at the same geographical location.

The cancer care pathways, as defined in 2009 by The Danish National Board of Health, determine the maximum time intervals for diagnostic processes and treatment as follows:

- Time from referral (time of diagnosis) to end of primary investigation = 28 days
- Time from end of primary investigation to first day of treatment = 14 days
- Time from referral (time of diagnosis) to first day of treatment = 42 days

Referral is defined as the date where the investigating department receives the referral.

End of primary investigation is defined as the date of decision on treatment.

First day of treatment is defined as the date of initiation of surgical, oncological, or radiological treatment, whichever comes first.

### The Danish Lung Cancer Register

Since the establishment in 2000, the Danish Lung Cancer Registry (DLCR) has accumulated data on all cases of lung cancer as reported from all of the more than 50 departments involved in the care of primary lung cancer patients in Denmark [1, 22]. Data are reported to the database when the diagnostic evaluation has been completed, and when a specific treatment has been finished. This registry information is then supplemented with data on the patient’s vital status retrieved from the Danish Civil Registration System, and pathology information related to the lung cancer case from the Danish Pathology Register. Since 2005 more than 90% of all Danish lung cancer patients have been included in the database. At present the database contains data describing waiting times, diagnostic procedures, staging, surgical procedures, complications, oncological treatment and survival on more than 45 000 patients [1]. The data needed in this study to describe potential delay and predictors of delay, are all contained in the database.

### Study population

Patients diagnosed with primary lung cancer from January 1st 2008 to December 31st 2012 were identified in the DLCR. Cases with a pathologically confirmed diagnosis of small cell lung cancer (SCLC) were excluded from the study. Also patients with incomplete data as regards date of referral to diagnosing hospital, date of diagnosis, date of referral to treating hospital and date of first treatment were excluded.

A total of 21 479 patients were identified in DLCR within the study period. 2841 of these patients had small cell lung cancer (SCLC). Another 1471 patients were excluded from the study due to undecided/unresolved pathology. 1805 patients did not have data on the primary investigation. In 180 cases data error occurred. Finally, data on the treatment were missing in 4328 cases. The final study population of 11 273 patients meets the inclusion criteria.

### Baseline characteristics

From the DLCR we obtained data on age at the time of diagnosis (day of referral to the diagnostic department), disease stage at the time of diagnosis, type of treatment, FEV1 (Forced expiratory volume in 1 second) and Eastern Cooperative Oncology Group (ECOG) performance status on the time of diagnosis and information about changing a hospital after the primary investigation. We included information on comorbidity for each patient up to 10 years before lung cancer diagnosis in the form of Charlson’s Comorbidity Index [23], using the Danish National Patient Register. Baseline patient characteristics are shown in Table 1.

**Table 1:** Baseline characteristics of the study population

		No transfer (%)	Transfer (%)	Total (%)	*P*-value
N		4434	6839	11,273	
Age^a^	<68	2068 (46.64)	3275 (47.89)	5343 (47.40)	0.198
	> = 68	2366 (53.36)	3564 (52.11)	5930 (52.60)	
Sex	Male	2299 (51.85)	3548 (51.88)	5847 (51.87)	0.976
	Female	2135 (48.15)	3291 (48.12)	5426 (48.13)	
Stage	0, I, II, IIIa	1611 (36.33)	3450 (50.48)	5061 (44.89)	<0.000
	IIIb, IV	2799 (63.13)	3347 (48.94)	6146 (54.52)	
	Missing	24 (0.54)	42 (0.61)	66 (0.59)	
Co-morbidity (CCI)	0	2333 (52.62)	3479 (50.87)	5812 (51.56)	0.156
	1	1829 (41.25)	2904 (42.46)	4722 (41.99)	
	>1	272 (6.13)	456 (6.67)	728 (6.46)	
Treatment	Curative	1287 (29.03)	3435 (50.08)	4712 (41.80)	<0.000
	Palliative	3147 (70.97)	3414 (49.92)	6561 (58.20)	
ECOG	0	1618 (36.49)	3068 (44.86)	4686 (41.57)	<0.000
	1	1705 (38.45)	2299 (33.62)	4004 (35.52)	
	>1	1111 (25.06)	1472 (21.52)	2583 (22.91)	
Year	2008	1259 (37.24)	747 (62.76)	2006 (100)	<0.000
	2009	1408 (33.65)	714 (66.35)	2122 (100)	
	2010	1452 (37.09)	856 (62.91)	2308 (100)	
	2011	1504 (40.48)	1023 (59.52)	2527 (100)	
	2012	1216 (47.36)	1094 (52.64)	2310 (100)	
FEV1 N (Mean, sd)		3842 (1.83,0.85)	6363 (1.96, 0.89)	10,205 (1.91,0.87)	<0.000
Days from referral to treatment Mean(sd)		35.59 (14.58)	40.19 (15.68)	38.38 (15.42)	<0.000
Days from referral to diagnosis Mean (sd)		20.26 (11.59)	22.26 (12.43)	21.47 (12.13)	<0.000
Days from diagnosis to treatment Mean (sd)		15.33 (9.70)	17.93 (11.09)	16.91 (10.64)	<0.000

^a^68 is the mean age of the patient population

### Statistical methods

Cox proportional hazard multivariable regression was used to assess the impact of transfer after the end of primary investigation on delay in the diagnosis, waiting time from end of primary investigation to treatment and total time from referral received to first day of treatment adjusting for age, sex, stage, type of treatment, Charlson’s Comorbidity Index (CCI), performance status (ECOG), FEV1, and year of diagnosis. Although Cox regression may indicate that the transfer has an impact on the delay in diagnosis, waiting time or total time this impact may be clinically insignificant. To find out the impact of transfer, on the accordance with recommendations, we used a multivariable logistic adjusted for age, sex, stage, treatment, CCI, ECOG, FEV1 and year of diagnosis. The following variables thus act as confounders in a prediction model: *Age*, low age as age being lower than 68 years, which is the mean age for the patient population and high age as being higher than the mean age for the patient population; *Sex*, *Comorbidity* (Charlson Comorbidity Index score, CCI): 0 as CCI score of 0, 1 as CCI score of 1; and 2 as CCI score >1, and *Clinical tumor stage*: 0 as clinical stage is equal to 0, I, II or IIIa and 1 as clinical stage is equal to IIIb or IV; *ECOG*: 0 as ECOG score of 0, 1 as ECOG score of 1, and 2 as ECOG score >1, and *FEV1* as a continuous variable.

## Results

A total number of 11 273 cases with NSCLC and complete data on both primary investigation and treatment were included in the study within the study period from 2008 to 2012. As regards the variables age, sex and co-morbidity there were no substantial differences between the “No-transfer” and the “Transfer” groups.

In the variable “stage” there is significantly more patients with disease stage 0-IIIa in the Transfer-group (50.48%) compared to the No transfer-group (36.33%). Conversely, there are more patients with disease-stage IIIb-IV in the No transfer-group (63.13%) compared to the Transfer-group (48.94%). This also applies for the variable “treatment” where 50.08% of the patients in the Transfer-group are treated with curative intent versus 29.03% in the No Transfer-group. Curative intent is defined as surgical resection or radiotherapy (stereotactic therapy or more than 50 GY). In the variable “year” the development over time is shown. In 2008 62.76% of the patients were in the No transfer-group and 37.24% in the transfer-group. The numbers for 2012 were 52.64 and 47.36%, respectively (Table 1).

**Fig. 1:**
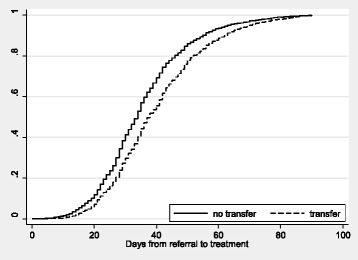
Kaplan-Meier estimates for “transfer” and “no-transfer” groups in total time from referral to first day of treatment (in days)

Based on unadjusted data, the total time from referral to first day of treatment for the two groups is illustrated in Fig. 1.

Table 2 shows time to event as a result of the Cox regression analysis.

**Table 2:** Results of Cox regression analysis

	Time from referral to end of primary investigation	Time from end of primary investigation to treatment	Total time from referral to treatment
	HR (CI 95%)	HR (CI 95%)	HR (CI 95%)
Transfer (yes vs no)	1.00 (0.93;1.08)	0.81 (0.68;0.96)^a^	0.84 (0.77;0.92)^a^
Age (high vs low)	0.89 (0.86;0.92)^a^	0.96 (0.91;1.01)	0.90 (0.87;0.94)^a^
Sex (males vs females)	1.00 (0.95;1.06)	1.03 (1.01;1.05)^a^	1.02 (0.99;1.06)
Stage (high vs Low)	1.12 (1.08;1.16)^a^	1.04 (1.00;1.08)	1.43 (1.30;1.56)^a^
Treatment (cur vs pal)	1.32 (1.24;1.41)^a^	0.86 (0.78;0.95)^a^	1.11 (1.00;1.24)
CCI (increasing)	0.93 (0.92;0.95)^a^	0.94 (0.91;0.98)^a^	0.91 (0.88;0.93)^a^
ECOG (increasing)	1.04 (1.01;1.08)	0.98 (0.96;1.00)	1.01 (0.99;1.04)
FEV1 (increasing)	0.99 (0.95;1.03)	1.03 (0.99;1.06)	1.02 (0.99;1.05)
Year (increasing)	0.97 (0.93;1.02)	1.15 (1.08;1.21)	1.08 (1.02;1.14)

^a^significance at 95% level

Hazard Ratio (HR)

- of 1 reflects that patients in the two groups analysed are diagnosed and treated within the same time
- below 1 reflects that patients in one group waits longer than the other (e.g. transfer vs no-transfer)
- above 1 reflects that patients in one group wait for a shorter time than the other.

Table 3 shows the odds of being diagnosed and treated in accordance with the maximum time intervals, adjusted for relevant variables.

**Table 3:** Results of Logistic Regression Analysis

	Time from referral to end of primary investigation ≤ 28 days	Time from end of primary investigation to first day of treatment ≤ 14 days	Time from referral to first day of treatment ≤ 42 days
	OR (95% CI)	OR (95% CI)	OR (95% CI)
Transfer (yes vs no)	0.82 (0.74;0.92^a^)	0.66 (0.61;0.72^a^)	0.63 (0.58;0.69^a^)
Age (high vs low)	0.85 (0.76;0.94^a^)	0.91 (0.83;0.99^a^)	0.78 (0.71;0.86^a^)
Sex (males vs female)	0.92 (0.83;1.03)	1.05 (0.96;1.14)	1.02 (0.93;1.12)
Stage (high vs low)	2.08 (1.84;2.37^a^)	1.17 (1.05;1.30^a^)	2.02 (1.81;2.25^a^)
Treatment (cur vs pal)	1.68 (1.47;1.91^a^)	0.72 (0.65;0.81^a^)	1.23 (1.10;1.38^a^)
CCI (increasing)	0.83 (0.77;0.91^a^)	0.91 (0.85;0.97^a^)	0.84 (0.78;0.90^a^)
ECOG (increasing)	1.08 (1.03;1.14^a^)	0.94 (0.90;0.98^a^)	1.00 (0.96;1.05)
FEV1 (increasing)	0.94 (0.88;1.00)	1.09 (1.04;1.15^a^)	1.03 (0.98;1.09)
Year (increasing)	0.98 (0.94;1.01)	1.29 (1.26;1.33^a^)	1.17 (1.05;1.20^a^)

^a^significance at 95% level

Results of the Cox regression analyses show that the time period from the end of the primary investigation to treatment for the patients, who were transferred, are significantly longer (HR = 0.81 with CI 95% (0.68; 0.96)) than the no-transfer patients, and that the total time from referral to treatment is also longer for these patients (HR = 0.84 with CI 95% (0.77; 0.92)). Results of the logistic regression analyses show that the odds of ending a primary investigation in 28 days from referral is significantly lower for the patients who were transferred (OR = 0.82 CI 95% (0.74; 0.92)), as well as the odds of getting treatment in 14 days after the end of primary investigation (OR = 0.66 CI 95% (0.61; 0.72)), and getting treatment in 42 days from the referral (OR = 0.63 CI 95% (0.58; 0.69)).

Both analyses show a slight effect of age on all time intervals. This indicates that patients above 68 years’ experience longer time from the end of the primary investigation to treatment and longer total time than younger patients. Gender does not seem to have an effect on the defined time intervals. Women wait marginally longer than men for treatment after the end of the primary investigation, but gender has no effect on the chance of being diagnosed and treated on time.

Overall patients with a high stage of disease have a significantly faster course of investigation as well as a significantly shorter time from referral to treatment (Table 2). Co-morbidity has an impact on all three time intervals (Table 2) where patients with high CCI-scores wait significantly longer for diagnosis and treatment than patients with low CCI-scores. Patients treated with curative intend experience significantly shorter time between referral and end of primary investigation compared to patients with palliative treatment (Table 2). On the other hand they wait longer for treatment after the end of the primary investigation. The difference between the two groups is somewhat equalized in the total time from referral to treatment. For all time intervals for performance status (ECOG), no difference was detected (Table 2). FEV1 as well as calendar year has no effect on any of the stated time intervals.

Results in Table 3 show that patients with a high stage of disease compared to low stage of disease has a substantially higher chance of being diagnosed and treated in time. The same tendency is shown for increasing CCI-score and for patients treated with curative intend. For performance status (ECOG), it seems that high performance status (low ECOG-score) increases the chance of being diagnosed within the acceptable timeframe and it seems to reduce the chance of getting treatment in time. The ECOG-level has no impact on compliance with the total time interval. Calendar year and diagnosis has no effect on the total length of diagnostic work-ups. Increasing FEV1 increases the chance of being diagnosed within the acceptable timeframe. Calendar year has a positive effect on the chance of getting treatment on time.

## Discussion

In Denmark, National Cancer Plans and Cancer Care Pathways determine acceptable time thresholds for diagnosis and treatment of lung cancer, but even though these plans where implemented as early as 2007 exceedance of time thresholds still is a problem nationwide. In this study, we wanted to investigate the impact on delay when transferred between hospital units, from referral to diagnosis and treatment in patients with NSCLC in Denmark.

The results show that transfer after finished primary investigation causes delay in the time interval from finished primary investigation to treatment and in the total time from referral to treatment, but does not affect the time from referral to the end of primary investigation. Furthermore, data shows that the odds of being diagnosed and treated within the maximum time intervals are negatively affected by transfer.

Transfer as a predictor for delay is not commonly described in the literature; only a few papers deal with how organizational factors influences delay. Schultz EM et al. examine the effect that institutional structures and processes of care have on delay, but the results are unequivocal [24]. Another study showed that the higher the number of hospitals visited before confirmed diagnosis the longer the delay [25]. In a comprehensive study of trends and predictors for delay in cancer surgery, approximately 55,000 patients with lung cancer were included. The study showed that patients diagnosed and treated at different hospitals had a 70% higher risk of exceeding the limit of time to treatment within 30 days of diagnosis compared to patients diagnosed and treated at the same hospital. Literature, however, tends to disagree on the conclusions on the impact of transfer [17, 26].

There does not seem to be a clear explanation of why transfer should cause delay. In fact, the very explicit and detailed description of the components and steps in the Danish cancer care pathways should prevent any excess delay and also avoid variation in the delay between hospitals on a national basis. The immediate and intuitive explanation would be that transfer between hospitals results in additional communication and administrative procedures that could slow down the patient pathway. Therefore great effort has been conducted to eliminate the possible negative effect of transfer conditional to administrative procedures. Thus all departments regardless of geographical distances have now been allocated to multidisciplinary teams (MDT) and all patients are discussed through video link on the MDT conferences with representatives from all relevant specialties present. These changes have been implemented during recent years and the effect still has to be evaluated.

Regarding the other examined variables related to personal and clinical circumstances of the individual patient, the results seem plausible from both clinical experience and the available literature.

Advanced age, high co-morbidity and low performance status seem to be associated with delay from referral to diagnosis and in some cases also from diagnosis to treatment and total time [6, 16, 26–29].

The two groups in our study differ in two important aspects. The No Transfer-group had a higher proportion of patients in high stage and accordingly a lower treatment rate. This reflects the organizational structure of the Danish health care system dealing with lung cancer, where a relatively high number of departments perform investigational procedures to diagnose lung cancer and a minor number of departments perform treatment. The patient pathway in patients with high stage and low performance is therefore terminated before referral in departments without treatment possibilities while, on the other hand, patients with low stage and high performance are referred to departments with treatment opportunities and these departments will thus accumulate low stage patients.

Patients diagnosed with a low stage tumour wait longer from referral to treatment than patients with a high stage tumour. This is a well-known observation related to the more complex and extensive diagnostic procedures these patients have to go through to define the treatment adapted to their condition. A low stage of disease at diagnosis can lead to delay in time to treatment as it often requires additional investigations to determine the patient’s suitability for treatment [6, 17–19, 30, 31].

Ellis and Olsson both found that complex diagnostic procedures cause delay with many different specialists and many steps from diagnosis to treatment causing delay [10, 21].

Whether delay in diagnosis and treatment influences the prognosis and mortality is debated in the literature. Several studies examine the effect of delay in the diagnosis and/or treatment on prognosis but an unequivocal association has not been demonstrated. On the contrary, the results seem to be both mixed and even paradoxical [1, 9, 10, 13, 26, 32–37].

Even if, at present, there is insufficient and inconclusive results concerning the association between delay and the prognosis of lung cancer, it still seems relevant to address the excess waiting time for diagnosis and treatment. It cannot be dismissed that delay affects the prognosis and delay for diagnosis and treatment can in any case be psychologically stressful for both patients and relatives [38–40]. Our study shows a significant difference between the groups and even though this is of minor quantitative magnitude, in this light it still might be of clinically significant importance and the results should warrant further focus on the limitation of the effects of transfer between hospitals.

In a recent study, Torring et al. highlighted the complex nature of the waiting time paradox, where the difference in the prognosis of patients works as a confounding factor when studying the waiting times, since priority in the diagnosis and treatment is given to patients who appear to be more ill. This confounding by indication could affect our results and explain some of our results concerning the differences between stages [41]. In our study we have found that the likelihood of increased time for diagnostic evaluation and time to treatment is increased even after stratification for low versus high stage (data not shown), suggesting that the confounding effect of stage per se is limited.

Despite this study being the largest in Denmark addressing the subject, the study has some limitations. It is a register based retrospective study and consequently issues concerning data completeness, accuracy of dates and algorithms is of importance [1], and furthermore a substantial number of cases are missing. Of the 17 167 patients with NSCLC who were potential for inclusion in the study, 6133 patients (36%) were excluded because of missing data on either the primary investigation or the treatment. Most likely the lacking data is due to failure to report. The question is whether there is a systematic underreporting and how that might influence the results. It is, however, our conclusion that the number of included patients and our assumption of an equal distribution of the Transfer and No transfer patients between the reported and missing cases, makes the results valid and useful.

## Conclusion

We conclude that transfers between hospital units in the patient course from referral to treatment might cause significant delay. The difference between no-transfer and transfer patients persists after adjusting for known predictors of delay. It remains to be investigated whether this prolonged time period has any impact on prognosis or mortality. Despite these limitations our study indicates that transfer between hospitals might have a significant impact on the delay, and this should be taken into account when planning the patient pathway. Caretakers should consider a primary transfer of patients initially thought to be suitable for treatment with curative intent and this policy should be included and defined in guidelines and definitions of patient pathways.

## Abbreviations

CCI: Charlson’s Comorbidity Index
DLCR: Danish Lung Cancer Registry
ECOG: Eastern Cooperative Oncology Group
FEV1: Forced expiratory volume in 1 second
NSCLC: Non-Small Cell Lung Cancer
SCLC: Small Cell Lung Cancer
